# Cultivar Variation in Tomato Seed Coat Permeability Is an Important Determinant of Jasmonic Acid Elicited Defenses Against Western Flower Thrips

**DOI:** 10.3389/fpls.2020.576505

**Published:** 2020-11-11

**Authors:** Sanae Mouden, Iris F. Kappers, Peter G. L. Klinkhamer, Kirsten A. Leiss

**Affiliations:** ^1^Plant Science and Natural Products, Institute of Biology, Leiden University, Leiden, Netherlands; ^2^Business Unit Horticulture, Wageningen University and Research, Bleiswijk, Netherlands; ^3^Laboratory of Plant Physiology, Wageningen University and Research, Wageningen, Netherlands

**Keywords:** induced resistance, seed receptivity, elicitor, *Frankliniella occidentalis*, scarification, *Solanum lycopersicum*

## Abstract

Induction of defenses is one of the most widely accepted eco-friendly approaches for management of pests and diseases. Seeds are receptive to resistance-inducing chemicals and could offer broad-spectrum protection at the early stages of development. However, seed treatment with elicitors has previously been shown to differentially influence induced defense responses among cultivars and thus, could hamper commercial exploitation. In this context, the objective of the present study was to evaluate the genotype-dependent ability of jasmonic acid (JA) to induce resistance against western flower thrips (WFT) at the seed stage. We examined the variation in inducibility of resistance in eight commercial tomato cultivars. Causal factors accounting for discrepancies in JA-induced responses at the seed stage were phenotypically and biochemically evaluated. Seed receptivity to exogenous JA appeared to be cultivar dependent. Thrips associated silver damage was only reduced in JA seed-treated plants of cultivar Carousel. Enhancement of resistance, was not associated with activation of defense-related traits such as polyphenol oxidase activity (PPO), trichomes or volatiles. Sulfuric acid scarification, prior to JA seed incubation, significantly augmented the embryonic responsiveness to JA in cv. Moneymaker without an adverse effect on growth. Hence, these results support the hypothesis that seed coat permeability is a key factor for successfully inducing JA mediated thrips defenses. The outcome of our study is of translational value as it creates opportunities for the seed industry to perform pre-treatments on non-responsive cultivars as well as for tomato breeding programs to select for genetic traits that affect seed permeability.

## Introduction

Plants are continuously under attack by a variety of pests and pathogens. Besides constitutive defenses, plants may also deploy inducible defenses that are activated in response to an attack. The expression of inducible defenses is primarily mediated by endogenous signaling molecules, among which the phytohormone jasmonic acid (JA) plays a key role in resistance against herbivorous arthropods and necrotrophic pathogens (Howe and Jander, 2008; Pieterse et al., 2012). For several decades it is known that activation of the JA signaling pathway is characterized by induction of multiple defensive traits including secondary metabolites (Bennett and Wallsgrove, 1994; War et al., 2012), proteins (Farmer and Ryan, 1990; Thaler et al., 1996), leaf trichomes (Boughton et al., 2005) as well as indirect induction mechanisms such as the production of plant volatiles (Ament et al., 2004; Arimura et al., 2005). Plant signals responsible for inducing resistance are highly conserved among plant species. Accordingly, artificial manipulation of these JA-associated defenses by natural or synthetic elicitors has proven to confer enhanced resistance against multiple insects and diseases and is, therefore, regarded as a valuable component in pest management programs (Thaler, 1999; Vallad and Goodman, 2004; Walters et al., 2013; Steenbergen et al., 2018).

Despite the long-standing importance of jasmonates in plant resistance, commercial applications have up to date met with rather limited success, as induced defenses are often associated with yield penalties (Cipollini et al., 2003; Walters and Heil, 2007). However, JA application does not necessarily lead to direct activation of defense mechanisms, as it may also condition a plant for boosted responses to future attacks, a phenomenon commonly known as priming (Conrath et al., 2006; Martinez-Medina et al., 2016; Mauch-Mani et al., 2017). The prospects of broad-spectrum enhanced resistance provided by priming has led to increased interest in the development of agents which can mimic natural inducers of resistance, particularly because priming is expected to cause a positive cost-benefit balance in times of stress (Van Hulten et al., 2006).

The vast majority of studies on JA induction focused on foliar application and root drenches, whereas the use of elicitors in seed treatments has been investigated less vigorously despite the fact that seedlings and young juvenile plants can be particularly more vulnerable to herbivory (Moles and Westoby, 2004; Barton and Hanley, 2013; Ochoa-López et al., 2015). Worrall et al. (2012) demonstrated that tomato seeds are receptive to plant defense activators and, thus, are able to activate herbivore resistance responses by stimulating the plant’s own defense mechanisms. Plants grown from JA-treated seeds exhibited long-lasting increased resistance against herbivores of multiple arthropod taxa, persisting over 8 weeks, without concurrent impact on plant growth. These findings were corroborated by Paudel et al. (2014) and Strapasson et al. (2014), who demonstrated that seed soaking of tomato cultivars Micro-Tom and Santa-Clara with methyl jasmonate (MeJA) induced durable resistance against herbivory by *Helicoverpa zea* and *Tuta absoluta*, respectively. Hence, seed treatments can be particularly useful in activation of induced resistance, especially for crops which are likely to face pest and pathogen attack early in the growing season. Intriguingly, JA seed treatment of the tomato cultivar “Moneymaker” failed to induce resistance against *Tetranychus urticae*, whereas a significant reduction in oviposition on JA-seed treated plants was observed in the tomato cultivar “Carousel” (Smart et al., 2013). Whilst the mechanisms behind these differential responses remain unclear, differences in the availability of inducible defense genes have been suggested to play an important role. The phenomenon of genetic variation in inducibility of resistance has been demonstrated in numerous studies, however, the majority of these considered variation in plant responsiveness caused by pests and pathogens rather than variation in defense responses elicited by chemicals (Degen et al., 2004; Sharma et al., 2010; Bruce, 2014). Alternatively, genetic factors underpinning seed coat permeability may also explain variations in elicitor induced responses across cultivars. The existence of a semi-permeable layer has been extensively reported in several species, including tomato, yet its importance for seed treatments has rarely been addressed (Salanenka and Taylor, 2011). Seed uptake of JA is pivotal and insufficient penetration into the seed would fail to activate defenses. For potential inclusion of elicitors in integrated pest management (IPM) programs it is, therefore, of importance to understand how these factors potentially affect plant resistance mechanisms.

To date, little is known about differences in elicitor-mediated induced resistance during the seed stage. This prompted us to evaluate several key questions concerning the inducibility of resistance against western flower thrips (WFT; *Frankliniella occidentalis*) by exogenous application of JA at the seed stage. Western flower thrips is one of the most economically important pest insects worldwide (Mouden et al., 2017). This polyphagous insect causes extensive damage in agriculture through feeding, oviposition as well as through the transmission of tospoviruses. Artificial induction of JA-mediated defenses has been reported to increase resistance to thrips in cotton (Omer et al., 2001), Chinese cabbage (Abe et al., 2009), and tomato (Escobar-Bravo et al., 2017).

In the present study, we addressed the following questions: (i) can tomato seed treatments with JA confer resistance to WFT? (ii) if so, are the defense responses against thrips elicited by JA seed soaking cultivar dependent? (iii) what mechanisms underlie this elicitor-mediated resistance? and (iv) can tomato seed scarification, prior to exogenous JA seed treatment, augment thrips resistance in cultivars that are non-responsive?

## Materials and Methods

### Seed Treatments and Growth Conditions

Seed treatments were performed as previously described by Worrall et al. (2012) with slight modifications. Briefly, a jasmonic acid stock solution (0.476 M) was purchased from Cayman Chemical Company (United States) containing 1 g JA in 10 ml absolute ethanol which was diluted to appropriate concentrations prior to use. Tomato seeds were soaked in an excess amount of freshly prepared dilutions of the JA stock solvent and incubated at 4°C in a rotary shaker for 24 h to facilitate the penetration of JA into the seeds. Control seeds were soaked in a mock solution consisting of aqueous ethanol under the same conditions. Following incubation, seeds were thoroughly washed in aqueous ethanol followed by sterilized MilliQ water. Subsequently, seeds were randomly sown in plastic trays filled with potting soil (Horticoop, Lentse Potgrond, Netherlands) and placed in a climate room provided with 113.6 μmol photons m^–2^ s^–1^ of photosynthetically active radiation (PAR) and a photoperiod of 16/8 h light/dark, at 20°C and 70% RH. 10 days later, seedlings were transplanted into individual plastic pots (11 × 11 × 12 cm) and grown under the same conditions for a period of 4 weeks. Pots were watered when needed.

### Non-choice Whole Plant Thrips Bioassay

A non-choice whole plant bioassay was used to evaluate tomato resistance against WFT (Leiss et al., 2009). Four-week old tomato plants were individually placed in thrips-proof cages consisting of a perspex cylinder (50 cm height, 20 cm diameter) closed at one end with a displaceable ring of nylon gauze of 120 μm mesh size. Plants were randomly placed in a climate-controlled growth chamber at a constant temperature of 25°C, a photoperiod of 16L:8D and 70% RH. For thrips infestation 20 adult thrips (18 females and 2 males) were collected using a mouth-operated aspirator and were then released inside the cage. Western flower thrips were obtained from a colony reared on chrysanthemum flowers (cultivar Euro Sunny) maintained in a climate room at 25°C and 70% RH. Seven days after infestation, thrips feeding damage, hereafter referred to as “silver damage” was evaluated by visually scoring all leaves. Silver damage was expressed as total damaged leaf area in mm^2^.

### Comparison of JA Seed and Foliar Treatments

To explore the potential of JA as an active inducer of systemic resistance at the seed stage, we aimed to determine its capacity to induce resistance to WFT and the consequent implication onproduction of defensive response markers (i.e., polyphenol oxidase) in tomato. For this, the systemic nature of JA-induced protection against WFT was evaluated by comparing the efficacy of two application methods namely: foliar spray and seed soaking. Seeds of the tomato cultivar Virona (provided by Incotec International BV, Enkhuizen, Netherlands) were immersed in JA solutions at concentrations ranging from 0.1 to 5 mM JA, under the same conditions as described above. Seeds treated with aqueous ethanol were used as controls. For foliar application, treatment concentrations and controls were equal to those used in seed treatments. To avoid direct toxic effects of foliar applied JA, 2 ml aliquots were uniformly sprayed 72 h prior to thrips infestation. Four weeks old plants were subjected to a non-choice whole plant thrips bioassay (*n* = 10). Plant growth parameters were measured prior to infestation, to evaluate potential growth effects. Furthermore, we explored plant defense responses triggered upon JA treatments by measuring the activity of the defense-related marker protein polyphenol-oxidase in non-infested plants (PPO; *n* = 4).

### Genotypic Variation in JA Seed Receptivity

To determine responsiveness of tomato seeds to JA induced defenses against WFT, an additional eight different cultivars were evaluated. Tomato cultivars, Carousel, 72-323, Mahitos untreated, Mahitos commercial (hydro-primed seeds), Brioso, Trovanzo and Raymos were kindly provided by Rijk Zwaan (de Lier, Netherlands), whereas cv. Moneymaker was obtained from Pieterpikzonen BV (Luinjeberd, Netherlands). The selected commercial tomato cultivars varied in fruit type, with the majority of cultivars producing classic round tomatoes. Brioso, on the other hand, was characterized as cocktail-type whereas, Mahitos produces beef tomatoes. Four weeks old plants, grown from JA (3 mM) or mock treated seeds, were subjected to a non-choice whole plant thrips bioassay (*n* = 10) or sampled for polyphenol oxidase (PPO) activity analysis (*n* = 7–8).

### Differences in Water Permeability Among Seeds of Different Cultivars

To explore whether permeability differences among cultivars contribute to the variability in JA responsiveness, an imbibition test was carried out by monitoring the fresh seed mass increase in the eight selected cultivars. Hundred seeds, separated into four replicates of 25 seeds each, were used for this test and weighed to the nearest 0.0001 g. Seeds were fully submerged in a freshly prepared solution of 3 mM jasmonic acid and kept at 4°C. The seeds were blotted and weighed at indicated time intervals for 24 h. The percentage mass increase (fresh mass basis) for each interval was calculated as % increase in mass = [(W_i_−W_*d*_)/W_d_] × 100, where W_i_ = mass of imbibed and W_d_ = mass of dry seeds (Baskin et al., 2004).

### Improvement of JA Seed Receptivity by Scarification

With the aim to explore whether seed scarification could augment JA receptivity at the seed stage, we evaluated the effect of scarification on JA-induced defenses against WFT, using tomato seeds of one of the non-responsive tomato cultivars. Initially, three scarification methods were evaluated in cultivar Virona. For mechanical scarification, seeds were individually scarified by a razor blade. Chemical scarification was performed with 10% sulfuric acid. A non-specified commercial scarification method was carried out by the seed company Incotec International BV (Enkhuizen, Netherlands). Non scarified seeds served as control. Preliminary experimental results demonstrated that chemical scarification with sulfuric acid, prior to JA soaking, was the most potential method to improve thrips resistance in cv. Virona. In a follow-up experiment, seeds of the unresponsive tomato cultivar Moneymaker and the JA-seed receptive cultivar Carousel were used to study the effect of scarification on JA-inducibility. Tomato seeds were chemically scarified with 10% sulfuric acid (H_2_SO_4_) for 5 min and shaken on a platform shaker at slow to moderate speed. After exposure, seeds were thoroughly washed with distilled water to remove residual sulfuric acid and subjected to JA (3 mM) soaking as previously described. Four weeks old plants were evaluated for thrips silver damage 7 days after infestation using a non-choice bioassay.

### PPO Activity

Polyphenol oxidase (PPO) activity was determined in non-infested plants by sampling one leaflet taken from the third leaf from the bottom, following the protocol described in Stout et al. (1998). In brief, 150 mg of fresh leaf material was flash frozen in liquid nitrogen and stored at −80°C until analysis for PPO activity. The enzyme was extracted by homogenizing ground plant material in 1.25 ml ice-cold potassium phosphate buffer (0.1 M, pH 6.8) containing 7% polyvinyl polypyrolidine, and 400 μl of 10% Triton X-100. Extracts were vortexed for 2 min and centrifuged at 11,000 × g for 10 min at 4°C. The supernatant was then collected as the crude enzyme extract. PPO activity was measured immediately after the extract was obtained. Activity was assayed by measuring the oxidation of chlorogenic acid as substrate in a reaction mixture containing 5 μl of the enzyme extract and 1 ml of 2.92 mM chlorogenic acid prepared in 0.1 M potassium phosphate buffer at pH 8.0. The increase in absorbance at 470 nm was measured spectrophotometrically (UV-1800 UV-VIS spectrophotometer, Shimadzu Europe GmbH, Duisburg, Germany) for 1 min. PPO activity was expressed as changes in absorbance values per min per gram of fresh weight.

### Determination of Type VI Glandular Trichome Density

In tomato, foliar application of JA or its volatile derivative methyl jasmonate has been reported to induce the production of type-VI leaf glandular trichomes (Escobar-Bravo et al., 2017; Chen et al., 2018). To investigate whether exogenous JA seed treatment also influenced physical leaf properties, we measured the density of type-VI glandular trichomes following the methodology described by Chen et al. (2018). The second terminal leaflet from the third youngest and fully expanded leaf was sampled in 4 weeks old plants grown from mock- and JA-treated seeds. A Leica stereomicroscope (MZ16, Leica Microsystems, Wetzlar Germany) equipped with a Leica DFC420 digital camera was used to take pictures on the adaxial surface at both sides of the main vein in the middle section of the leaflet. Trichomes were counted in an area of 12 mm^2^ using Fiji ImageJ software^1^ and expressed as the number of type-VI trichomes per centimeter square.

### GC-MS Analysis of Terpenes

Artificial induction of type-VI glandular trichomes and their associated volatiles has been related to increased levels of resistance against diverse herbivorous arthropods (Kang et al., 2010; Tian et al., 2012; Escobar-Bravo et al., 2017) Terpene production from type-VI trichome-derived leaf exudates was evaluated using the leaf dip method. This protocol was chosen because the terpenoid profile detected in individually collected type VI glands has been shown to be nearly identical to that observed with the leaf dip procedure (Kang et al., 2010, 2014). Four weeks after initial seed treatment, two leaflets belonging to the same leaf used for trichome density measurement, were sampled and leaf fresh weight was measured before extraction. Leaf exudates were obtained by dipping the leaf tissue in 2 ml of pentane. Following an incubation period of 2 min with gentle shaking, leaflets were discarded, and the extracts were analyzed by gas chromatography-mass spectrometry (Kappers et al., 2011). Two microliter of pentane extracts were splitless analyzed on a Hewlett–Packard GC-MS system consisting of a gas chromatograph (5,890 series II, Hewlett–Packard) equipped with DB-5MS column (30 m × 0.25 mm, 0.25 μm film thickness) and a mass-selective detector (model 5972A, Hewlett–Packard). Compounds were identified by comparing retention times and mass spectra with those of authentic standards and reported in Adams (2007). The area under the curve of selected compounds was calculated to be representative for the concentration.

### Statistical Analysis

Normal distributions were tested by Shapiro-Wilk tests and homogeneity of variances were determined by Levene’s tests. Means were analyzed using one-way ANOVA followed by Fisher’s least significance difference (LSD) *post hoc* test. When assumptions were violated, data transformations were performed or differences in means were compared using the nonparametric Kruskal-Wallis-test or Welch’s ANOVA. Data on number of leaves were analyzed by Kruskal-Wallis followed by Dunn’s test with Bonferroni correction for multiple comparisons. The effect of foliar JA application on silver damage and PPO activity were analyzed by Welch Anova followed by Games-Howell *post-hoc*. Generalized Linear Models (GLM) using linear distribution and identity link functions were used to analyze the effect of cultivar, seed treatment and their interaction on silver damage, PPO activity, number of trichomes, volatile emission and dry mass. Data on silver damage obtained from the scarification experiment were square root transformed and subsequently analyzed by GLM. Differences among groups were tested by Fisher’s LSD *post-hoc* test. All statistical analyses were conducted with SPSS v. 24 software (IBM; SPSS Inc., Chicago, IL, United States).

## Results

### Effect of Elicitor Application on Thrips Feeding Damage

**FIGURE 1:**
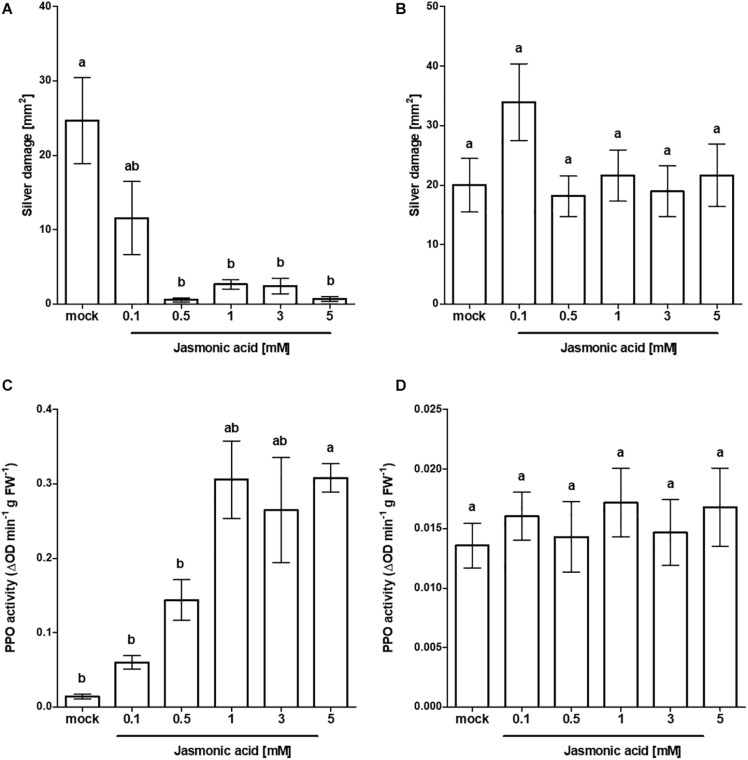
Effect of exogenous JA treatment on tomato resistance to WFT and JA-associated responses. Silver damage symptoms in tomato plants (cv. Virona) following **(A)** foliar application of JA or **(B)** JA seed treatment at different concentrations (*n* = 10). Four-week old plants were infested with WFT for 1 week or sampled for polyphenol oxidase (PPO) activity. PPO activity (*n* = 4) was measured in the third leaf from the bottom in **(C)** JA foliar treated plants or in **(D)** JA-seed treated plants. Data are presented as mean ± SEM. Different letters indicate significant differences among treatments (Welch ANOVA followed by Games–Howell at *P* < 0.05).

To explore the potential of JA as an active inducer of systemic resistance at the seed stage, we firstly compared seed responsiveness to foliar applied JA in tomato cv. Virona. Foliar application of JA caused a marked reduction in silver damage compared to mock-treated Virona plants (Welch’s *F*_(5, 24.27)_ = 7.53, *p* ≤ 0.001). With the exception of 0.1 mM JA, significant reductions in damage were observed, ranging between 53 and 97% (Figure 1A). The pattern of responses to thrips damage mirrored the induction of PPO (Figure 1C). Levels of PPO were greatly enhanced in response to foliar application of JA, showing a dose-dependent trend (Welch’s *F*_(5, 7.34)_ = 44.23, *p* ≤ 0.001). Next, we compared the efficacy of foliar applied JA in suppressing silver damage with the efficacy of seed applied JA (Figures 1A,B, respectively). In contrast to foliar application, exogenous application of different JA doses to the seed did not affect total silver damage (*H*(5) = 4.839, *p* = 0.436). The lack of increased resistance levels to thrips corresponded with the measured PPO levels (Figure 1D). PPO activity was not increased in JA seed treated plants (*F*_(__5,18)_ = 0.295, *p* = 0.909). Costs of foliar or seed JA treatment related to yield were investigated. The impact of seed soaking on plant performance were minimal. Whereas seed exposure to 5 mM JA adversely affected tomato growth, seeds pre-treated with intermediate concentrations of JA (1 and 3 mM) surprisingly caused a stimulatory effect on plant dry mass (Supplementary Table S1).

### Exploring JA Seed Receptivity Among Tomato Cultivars

**FIGURE 2:**
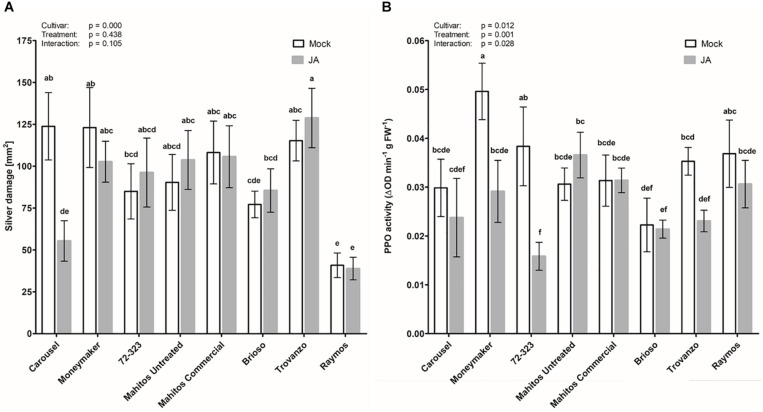
Effect of exogenous JA treatment on tomato resistance to WFT and JA-associated responses. Silver damage symptoms **(A)** and PPO activity **(B)** were measured in 8 tomato cultivars: Moneymaker, Carousel, 72-323, Mahitos untreated, Mahitos commercial, Brioso, Trovanzo and Raymos. Plants were subjected to mock or jasmonic acid (3 mM) seed treatment. When 4 weeks old, plants were exposed to a non-choice whole plant bioassay and infested with 20 adult thrips (*n* = 10). Silver damage symptoms were visually scored after 7 days of thrips infestation. **(B)** Polyphenol oxidase activity was measured in non-infested plants taking the third leaf from the bottom (*n* = 7–8). Data are presented as mean ± SEM. Different letters denote significant differences among groups as determined by GLM followed by Fisher’s LSD test (*P* < 0.05). The overall effects of cultivar, treatment and their interaction are indicated in each graph.

In the previous experiment, seed applied JA had no effect on silver damage in tomato cultivar Virona. Yet, application of JA as a seed soaking treatment in cultivar Carousel was capable of improving resistance against WFT (Supplementary Figure S1). Thus, we further examined variation among cultivars in the efficacy of thrips resistance induced by seed application of JA. Concomitantly, we evaluated whether seed applied JA affected the induction of the JA-associated marker polyphenol oxidase. Four weeks old plants of eight different tomato cultivars, grown from JA or mock treated seeds, were challenged with WFT (Figure 2A). Overall, JA seed treatment had no significant effect on silver damage, but symptoms were affected by plant genotype (GLM: Wald χ^2^ = 38.94, *p* < 0.001 for cultivar; Wald χ^2^ = 0.60, *p* = 0.438 for treatment and Wald χ^2^ = 11.87, *p* = 0.105 for the interaction). However, as in our preliminary experiment with Carousel, a strong reduction in silver damage symptoms was observed in JA seed treated Carousel plants. Such a reduction was absent in the remaining other seven cultivars. Silver damage symptoms of plants grown from untreated seeds, revealed that the eight cultivars varied inherently in susceptibility, from cultivar Raymos being moderately resistant with 40.6 ± 7.4 mm2 silver damage to cultivars Moneymaker and Carousel being highly susceptible with 123.1 ± 23.9 and 123.9 ± 20.1 mm^2^ damage, respectively. Furthermore, we observed that the seed treatment was not associated with a reduction in vegetative growth (Supplementary Figure S2). Cultivar, on the other hand, affected dry mass of seed treated plants (GLM: Wald χ^2^ = 89.44, *p* < 0.001 for cultivar; Wald χ^2^ = 0.175, *p* = 0.676 for treatment and Wald χ^2^ = 3.86, *p* = 0.795 for the interaction). To evaluate whether reductions in thrips feeding damage were associated with the JA signaling pathway, we measured PPO activities in JA-seed elicited plants and control plants in the absence of thrips. Seed applied JA had a significant effect on PPO levels (GLM: Wald χ^2^ = 18.09, *p* = 0.012 for cultivar; Wald χ^2^ = 10.86, *p* = 0.01 for treatment and Wald χ^2^ = 15.67, *p* = 0.028 for the interaction).the enhanced resistance following JA seed application in “Carousel” plants, however, was not associated with direct induction of plant defenses (Figure 2B).

### Differences Among Cultivars in Water Permeability

**FIGURE 3:**
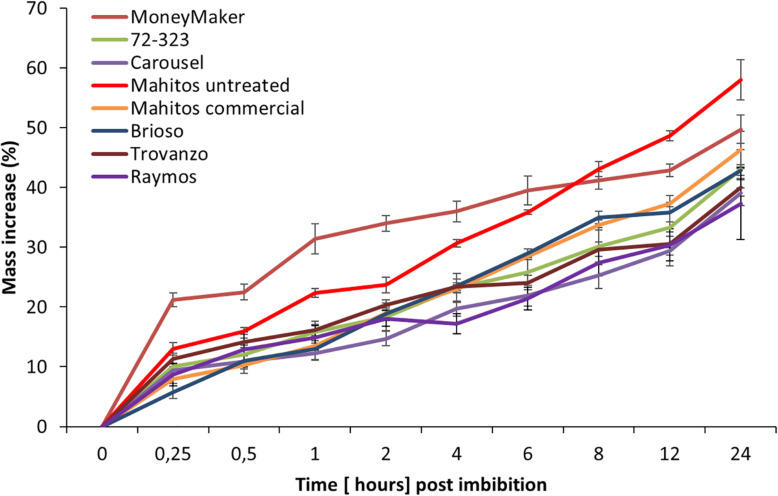
Imbibition curves of eight different tomato cultivars. The percentage of mass increase in weight was used as a measure of water imbibition. Different line colors correspond to different cultivars. Red, orange, green, blue, gray, brown yellow and purple correspond to the following cultivars; “Mahitos untreated,” “Moneymaker,” “Mahitos commercial,” “Brioso,” “72-323,” “Trovanzo,” “Carousel,” and “Raymos,” respectively.

To better understand the underlying mechanisms responsible for the differences in JA seed induced responses, we examined the eight tomato cultivars in relation to mass increase (Figure 3). The water imbibition pattern of Moneymaker showed a slightly curved pattern, while other cultivars had a more linear pattern. Within 1 h, water absorption differed strikingly among tomato cultivars (*F*_(7,16)_ = 5.376, *p* = 0.03). Moneymaker absorbed significantly higher amounts of water during this period in comparison to the other cultivars. However, no significant differences in final water uptake (i.e., after24 h) were observed between tomato cultivars (Welch’s *F*_(7, 6.66)_ = 1.11, *p* = 0.452). Nevertheless, Carousel, together with Raymos seeds hydrated at the slowest rate. Carousel gained only 39% by weight over the initial seed weight after 24 h soaking. The tomato cultivar Moneymaker, on the other hand, hydrated most rapidly and imbibed 10% more water than Carousel seeds in this time period (49.73 ± 2.35 vs. Carousel 39.02 ± 2.15). Moreover, we evaluated whether hydro-priming of commercial Mahitos seeds influenced subsequent water permeability. Seed priming is a commercially used pre-sowing technique which involves the uptake of water by the seed followed by drying to trigger pre-germinative events without protrusion of the radical through the seed coat. Such achieved physiological state enables seeds to germinate more efficiently (Taylor et al., 1992). Hydropriming of seeds had no significant effect on water imbibition percentage (46.30 ± 3.08 commercial vs. untreated i.e., unprimed Mahitos 58.02 ± 3.37).

### Phenotypic Characterization of Seed Receptivity

**FIGURE 4:**
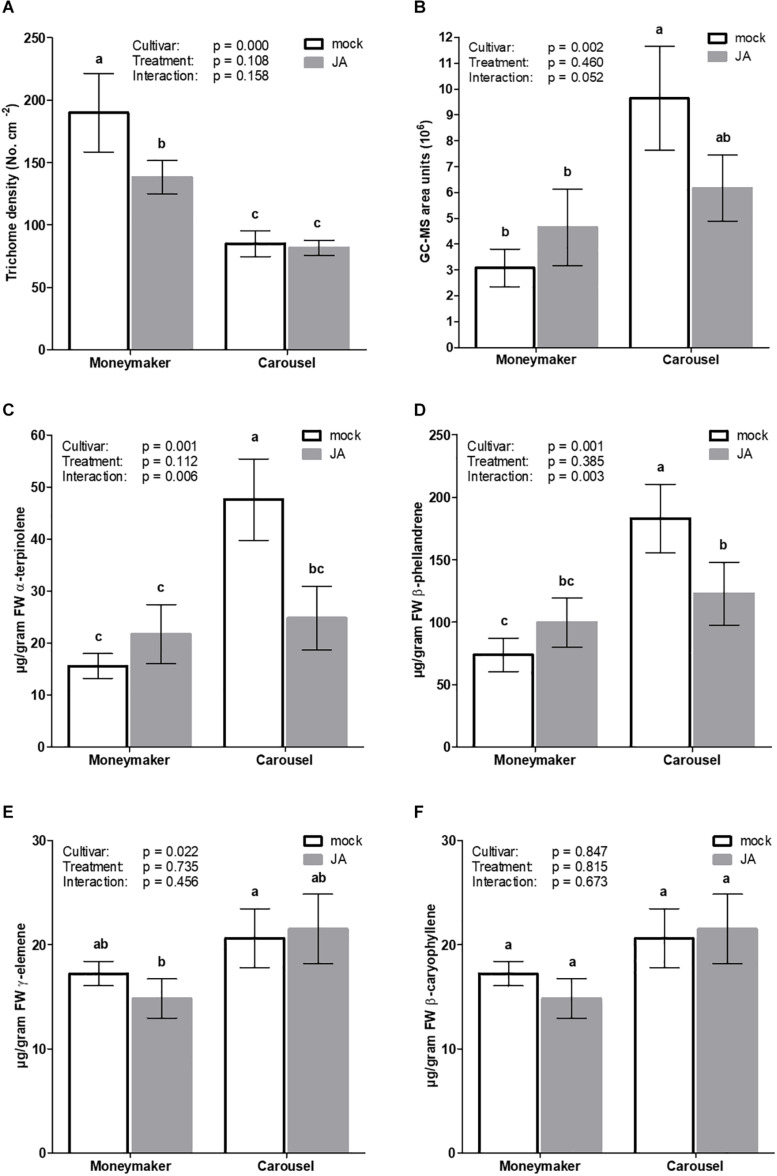
Effect of exogenous JA seed treatment on type-VI trichome density and trichome derived volatiles in uninfested tomato plants. Seeds of cultivar Moneymaker and Carousel were soaked in mock or 3 mM JA for 24 h. Four weeks after initial treatments, **(A)** type VI glandular trichome density (*n* = 10) was evaluated on the adaxial leafside of leaflets from the third youngest leaf. **(B)** total terpene content and content of monoterpenes **(C)** α-terpinolene, **(D)** β-phellandrene and sesquiterpenes **(E)** γ-elemene and **(F)** β-caryophyllene derived from leaf exudates of mock and JA-seed treated tomato plants (*n* = 5). Bars represent mean ± SEM. Different letters denote significant differences among groups as determined by GLM followed by Fisher’s LSD test (*P* < 0.05). The overall effects of cultivar, treatment and their interaction are indicated in each panel.

Based on the above results, Moneymaker and Carousel were chosen as “non-responsive” and “highly responsive” to JA seed soaking, respectively. With the aim to explore underlying differences in JA seed receptivity, vegetative plants resulting from JA soaked seeds from both cultivars were phenotypically characterized based on morphological and chemical criteria (Figure 4). Type-VI trichome density was significantly affected by cultivar (GLM; Wald χ^2^ = 22.260, *p* ≤ 0.001) but not by treatment (GLM; Wald χ^2^ = 2.576, *p* = 0.108). The cultivar by treatment interaction was not significant (GLM: Wald χ^2^ = 1.990, *p* = 0.158). The number of glandular trichomes in Carousel plants was 2.2-fold lower than in Moneymaker (Figure 4A). On the contrary, Carousel plants displayed significantly higher terpene content than Moneymaker plants (Figure 4B; GLM; Wald χ^2^ = 9.682, *p* = 0.002 for cultivar, Wald χ^2^ = 0.547, *p* = 0.460 for treatment and Wald χ^2^ = 3.778, *p* = 0.052 for interaction). Analysis of volatiles in leaf exudates revealed that cultivars differed in relative abundance of monoterpenes whereas no significant effect of treatment was observed (Figures 4C–E). Carousel plants produced significantly more α-terpinolene, β-phellandrene in comparison to Moneymaker plants (Figures 4C–E). Moreover, the results of the interactions suggested that seed treatment affected the emissions of α-terpinolene and β-phellandrene (GLM; Wald χ^2^ = 7.677, *p* = 0.006 and Wald χ^2^ = 4.711, *p* = 0.003 for interaction, respectively). Whilst upon JA seed soaking the monoterpenes α-terpinolene and β-phellandrene significantly decreased in Carousel, no significant effect was observed for JA seed treated Moneymaker plants (Figures 4C,D). Furthermore, no difference was observed for the sesquiterpene and β-caryophyllene (Figure 4F).

### Improvement of JA seed Receptivity by Scarification

**FIGURE 5:**
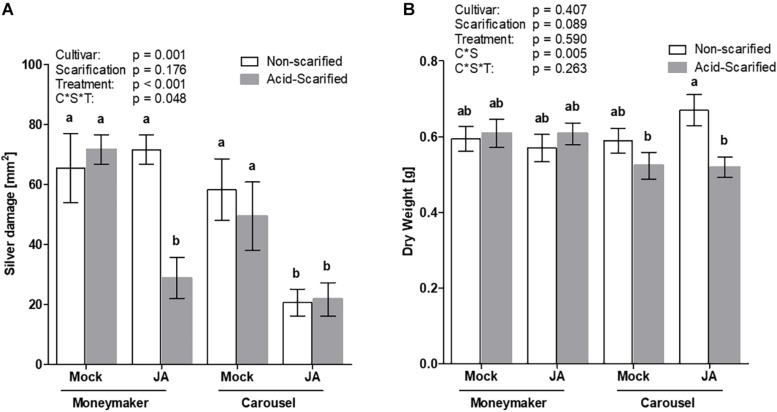
The effect of acid scarification on JA seed receptivity in Moneymaker and Carousel was measured by evaluating whole plant silver damage **(A)** and dry mass **(B)**. Seeds of the cultivar Moneymaker and Carousel were scarified with 10% sulfuric acid prior to seed treatments. Acid scarified seeds were soaked in 3 mM JA or 0.5% ethanol mock for 24 h. Bars represent mean ± SEM of 10 individual plants. Different letters denote significant differences among groups as determined by GLM followed by Fisher’s LSD test (*P* < 0.05). The overall effects of cultivar, treatment and the three way interaction are indicated in the graph. Two-way interactions were not significant except the interacting effect of cultivar * scarification on dry mass.

Systemic compounds must be able to permeate the seed coat and diffuse to the embryo in order to exert efficacy. To further demonstrate the importance of the seed coat several scarification methods were explored, prior to JA seed soaking, to determine whether embryonic responsiveness to JA could be augmented. Preliminary experimental results in one of the non-responsive tomato cultivars (Virona) demonstrated that chemical scarification with 10% sulfuric acid was the most potential method to improve JA seed receptivity with no adverse effects on the embryo and subsequent growth (Supplementary Figure S3). Therefore, acid scarification was applied to increase seed coat permeability of the non-responsive cultivar Moneymaker. The effect was compared with the highly responsive cultivar Carousel (Figure 5). The response to JA elicitation varied depending on cultivar, scarification and treatment (GLM; Wald χ^2^ = 3.896, *p* = 0.048 for three-way interaction). Seed applied JA reduced silver damage symptoms in Carousel plants, independent of acid scarification. Interestingly, acid scarification prior to JA seed treatment augmented seed receptivity in Moneymaker plants. Following JA soaking of Moneymaker seeds, whole plant silver damage was significantly reduced by 60% as compared to the acid-scarified mock treated control (Figure 5A). Whilst no negative effects of seed pre-treatments on dry mass were observed in Moneymaker, acid scarification significantly reduced dry mass in JA-seed treated Carousel plants (Figure 5B).

## Discussion

In the past decades, elicitor-mediated induced resistance has become one of the most challenging research areas in plant herbivory. Recent studies have shown that seeds are receptive to direct application of exogenous phytohormones without being constrained by the inherent costs of defense (Rajjou et al., 2006; Worrall et al., 2012; Haas et al., 2018). However, research on the beneficial effects of JA seed treatments are restricted to one or only few tomato cultivars (Smart et al., 2013). To further explore the potential of JA as an active inducer of systemic resistance, it was of interest to investigate whether its’ application at the seed stage could confer resistance against the economically important pest insect species WFT. Here, we show that in the tomato cultivar Carousel, JA seed treatment induced defenses against WFT. This result is in line with previous studies on other arthropod herbivores (Worrall et al., 2012; Smart et al., 2013). In addition, across eight tomato cultivars we demonstrated that seed receptivity of Carousel seeds appeared to be an exception, rather than the rule. Preliminary experiments with Moneymaker demonstrated that seeds of this cultivar were not receptive to JA-mediated defenses (Supplementary Figure S4). This is line with earlier findings obtained by Smart et al. (2013), who reported that Moneymaker plants lacked systemic induction of defense against the two-spotted spider mite *Tetranychus urticae* in response to JA seed treatment. These observations prompted us to investigate the differential seed receptivity responses of several commercial tomato cultivars.

Foliar JA-mediated herbivore resistance is a regulated at transcriptional and translational level. To this end, we first studied the influence of JA on elicitation of defense responses in tomato (cv. Virona) by comparing the efficacy of JA seed treatment with foliar application. Thrips resistance was differently affected by treatments. In contrast to foliar application of JA, which significantly reduced silver damage and caused a dose-dependent induction of the defensive enzyme polyphenol oxidase (PPO), JA seed treated plants failed to enhance resistance to thrips consistent with its inability to induce PPO activity. Interestingly, when we evaluated the tomato cultivar Carousel, JA seed treatment led to durable thrips resistance (Supplementary Figure S1). The question naturally arises as to “what features contribute to Carousels’ uniqueness?” A number of hypotheses have been put forth to account for differences in seed receptivity. Among these, genetic variation may play a major role in how plants respond to elicitor induced resistance (Sharma et al., 2010; Walters et al., 2013; Bruce et al., 2017; Goodwin et al., 2017, 2018). To the best of our knowledge, no studies have so far explored the underlying mechanisms accountable for variable JA induced responses at the seed stage. Subsequently, we decided to expand our range of cultivars to study variation in response to JA seed applied induced defenses. We observed that cultivar was a major factor in determining the efficacy of JA (Figure 2). Among the eight tomato cultivars, only Carousel seed-treated plants showed a significant decrease in silver damage without concurrent impact on vegetative growth compared to the mock control. Silver damage symptoms in mock-seed treated Carousel plants were, however, for an unknown reason twice as high as in the first experiment (Supplementary Figure S1 vs. Figure 2). Nonetheless, the percentage of symptom reduction was in both experiments approximately 50%. Increased resistance to WFT in JA seed-treated Carousel plants, however, was not associated with induction of PPO activities. Surprisingly, in the non-responsive cv. Moneymaker PPO levels were significantly reduced upon JA seed treatment suggesting that it may also exert also important physiological roles in addition to defenses. In contrast, readily measurable PPO activities were observed in foliar treated Carousel plants when JA was applied 3 days before thrips challenge (Supplementary Figure S1). One possible explanation for the lack of PPO induction is the time gap following JA seed treatment and PPO measurements. Following induction treatment, there is usually a set period of time required for the establishment of induced resistance. PPO activities may have increased after this lag period with a slow decrease thereafter. Furthermore, induced plant responses often decline with age and, in addition, are markedly influenced by leaf developmental stage (Cipollini and Redman, 1999; Chen et al., 2018). Perhaps, 4 weeks post treatment, when PPO activity was measured, induction of defense related enzyms, in the absence of thrips, might thus not be measurable anymore. Nonetheless, Paudel et al. (2014) demonstrated that the induction of plant defense against *Helicoverpa zea*, following MeJA seed treatment of tomato cultivar Micro-Tom, was correlated with increased PPO activity. Moreover, the effect of seed treatment was described to be considerably long lasting as 25- and 50-days old plants displayed significant increases in PPO levels. Rather than direct induction of defenses, it is plausible that JA seed treatments primed for enhanced defense reactions although this was not addressed by our experiments. So far, it remains inconclusive whether priming of JA responses functions as control mechanism underlying induced resistance in seed-treated Carousel plants. In addition to PPO activity, future experiments evaluating spatiotemporal distributions of JA marker genes following JA seed soaking could provide helpful information here.

### Seed Coat and Compound Characteristics

Seed coat permeability has also been postulated to contribute to discrepancies in JA-induced host responses (Smart et al., 2013). Tomato seeds are classified as having selectively permeable seed coat characteristics (Taylor and Salanenka, 2012). This semi-permeable layer, composed of suberin, is an important structure for restricting or impeding the penetration of some solutes into embryos during imbibition, while being permeable to water and gases (Beresniewicz et al., 1995b). To better understand the underlying mechanisms responsible for variable JA seed induced responses, we measured imbibition rates as a proxy to characterize seed coat permeability. In contrast to our expectations, water absorption characteristics did not strikingly differ among cultivars (Figure 3) while JA application had a significant effect on PPO. Intriguingly, Carousel seeds were shown to hydrate at the slowest rate and imbibed among the least amount of water after a soaking period of 24 h. contrast, the non-responsive cultivar Moneymaker hydrated most rapidly. Although not significant, Moneymaker imbibed more water than Carousel seeds. This suggests that discrepancies in JA induced responses at the seed stage are not directly explained by differences in seed coat permeability. The results of this experiment, however, must be interpreted in the context of a number of potential limitations. Firstly, the possible diagnostic value of seed coat permeability was assessed by comparing the water uptake which, by itself, does necessarily reflect uptake characteristics and subsequent bioavailability of JA. Jasmonic acid uptake by seeds is pivotal and insufficient penetration into the seed would fail to prime or induce defenses. Dissection and separation of seed tissues, followed by chemical extractions and quantification are a prerequisite to adequately evaluate whether induced host response variations arose from differences in JA uptake. We suggest follow-up studies to evaluate the uptake and metabolic fate of seed applied JA. Moreover, it is generally known that differences in seed coat anatomy play an important role in the process of water imbibition and that these differences relate to permeability (Koizumi et al., 2008). Physical properties of seeds such as surface area, sphericity and seed size, however, were not evaluated in the present study. In addition to structural differences, chemical compositions of seed covering layers may also provide a biochemical rationale for variations in uptake and bioavailability (Beresniewicz et al., 1995a; Vu et al., 2014). Comparison of soybean varieties, for example, revealed that the outer cuticle of non-permeable varieties contained disproportionally more hydroxylated fatty acids than permeable varieties (Shao et al., 2007; Qutob et al., 2008). The relative contribution of structural and chemical properties of the seed surface to understanding the seed responsiveness requires more in-depth analysis. Furthermore, seed coat compositions may differ from that of the embryo and, thus, can affect both permeability and affinity for a compound. Retention by the seed coat could result in suboptimal concentrations reaching the embryo which consequently, is unable to activate defense-related processes. Finally, a central question related to such differential embryonic responses is whether, and to what extent, seeds are transcriptionally or translationally responsive to exogenous plant hormones (Walker-Simmons, 1990; Liao et al., 2020).

Moreover, the physicochemical nature of JA in relation to seed coat permeability of different tomato cultivars could shed light on the lack of a systemic induced response to thrips. Non-ionic compounds that are moderately lipophilic in nature should be able to diffuse through the semi-permeable seed coat of tomato, whereas ionic compounds are blocked (Salanenka and Taylor, 2011). At a pH significantly below the pKa of the ionisable carboxylic center (pKa = 4.71; predicted by ChemAxon, Cambridge, MA, United States), the majority of JA occurs in its protonated or unionized form, whereas at a pH above the pKa, the ionized form predominates. It can be expected that a large proportion of JA, at a concentration of 3 mM, occurs in in a non-dissociated form and thus, theoretically could diffuse through tomato seed coats. Apart from the electrical charge, lipophilicity, measured by the octanol-water partition coefficient (Log Kow or Log P), forms another key determinant for the ability of a compound to penetrate the seed coat as it describes the affinity of compounds for the lipid phase of plant tissue (e.g., plasma membrane, waxes, cutin, suberin, etc.) (Braumann, 1986). Translocation of JA, a derivative of the fatty acid linolenic acid, could potentially be limited by the presence of suberin, which in general is well known to adsorb lipophilic compounds. The lipophilic behavior of JA is most dominant when the carboxylic group is unionized, as it becomes more soluble in the immiscible lipophilic solvent. Recently, Yang et al. (2017, 2018) studied the uptake and distribution of a series of nonionic fluorescent piperonyl amides in relation to their chemical lipophilicity (Log Kow) in permeable soybean and semi-permeable tomato and corn seeds. Contrasting trends in uptake between seed tissues were observed for soybean. In tomato, as in corn, the overall uptake pattern for seed covering layers was similar to that of the internal tissues (Yang et al., 2017). The maximum uptake for non-ionic compounds to diffuse to the embryo of tomato ranged between Log Kow 3–4. Interestingly, at optimal Log Kow <5% of the applied compound was measured in the embryo of tomato seeds. The predicted log P or Log Kow for JA is 2.41 (ChemAxon). This value lies in the lower range of permeability, and as such, the uptake efficiency in the embryo may, theoretically, even be <5%. Nonetheless, uptake efficiencies clearly varied among the three evaluated tomato varieties (Yang et al., 2017). Seed applied products with a high potency (i.e., require a lower effective concentration EC_50_) could, therefore, potentially compensate for their low permeability.

### Effect of JA Seed Treatment on Physicochemical Leaf Properties

We further investigated whether the enhancement of thrips resistance in the responsive cultivar Carousel was associated with induction of physical and chemical leaf properties. Type-VI glandular trichomes are controlled by the JA-pathway (Boughton et al., 2005; Maes and Goossens, 2010) and provide physical and chemical barriers which are known to produce and secrete diverse compounds affecting survival (Frelichowski and Juvik, 2001), growth (Kang et al., 2010) and fecundity (Bleeker et al., 2012) of many herbivorous arthropods. In tomato, artificial application of JA or its volatile derivative methyl jasmonate (MeJA) has been reported to induce the production of type-VI leaf glandular trichomes and volatile emissions (Escobar-Bravo et al., 2017; Chen et al., 2018). Carousel plants contained significantly less type-VI glandular trichomes than Moneymaker, however, we observed no significant effect of seed treatment on trichome densities. Intriguingly, total terpene content was significantly higher in mock seed treated Carousel plants, suggesting a relative decrease in terpene production per trichome in JA-seed treated plants. Furthermore, JA seed treatments markedly decreased the emission of the monoterpenes α-terpinolene and β-phellandrene in Carousel whereas, no significant effects were observed in JA seed treated Moneymaker plants. These findings are in agreement with Strapasson et al. (2014), who reported that MeJA seed treatment of cultivar Micro-Tom decreased volatile emissions, including that of β-phellandrene, in uninfested tomato plants. Consequently, manipulation of plant volatile emissions could contribute to the control of insect pests as herbivores rely on volatile cues for host location. Reduced levels of emitted volatile compounds involved in volatile-mediated foraging may lead to a decrease in host finding efficiency and, ultimately a lower number of offspring (Bruce et al., 2005; Proffit et al., 2011). On the contrary, foliar applications of JA or MeJA differentially affect volatile emissions. Significant increases in the emission of terpinolene and β-phellandrene were reported in cultivated (cv. Moneymaker) and wild-type (Castlemart) tomato plants and could potentially reinforce defenses as a result of their direct toxic or repellent potency (Bleeker et al., 2009; Escobar-Bravo et al., 2017; Chen et al., 2018). These intriguing findings suggests that indirect defenses can have opposite ontogenic trends.

### Acid Scarification Augments JA Seed Responsiveness

The phenotypical and bio-chemical analyses discussed above did not provide a causal explanation for the underlying characteristic low receptivity of tomato cultivar Moneymaker. Acid scarification prior to JA seed treatment was, therefore, evaluated to further demonstrate the importance of the seed coat permeability. Our results show that pre-treatment with 10% sulfuric acid significantly augmented receptivity in JA-seed treated Moneymaker plants and, hence suggest that the lack of defense induction was likely caused by seed coat impermeability. The observed enhancement in resistance is encouraging. It demonstrates the possibility to manipulate seed coat characteristics of non-responsive cultivars without the need of difficult and time-consuming breeding techniques. Further evidence that the lack of induced resistance to thrips was caused by impermeability comes from extended seed treatment experiments. Extended soaking (i.e., 6 days) of germinating tomato seeds (cv. Moneymaker) in JA solutions bypasses the limitations of reduced permeability of the tomato seed coat and increased the effectiveness of induced resistance. However, this approach is not without risk as prolonged soaking can cause serious negative effects on seed viability (Chen et al., unpublished). Luna et al. (2016) reported similar findings in Moneymaker using 1 mM β-aminobutyric acid (BABA) for durable induced resistance to *Botrytis cinera*. After a 7-days incubation period of seeds in BABA, lesion size was significantly reduced in 4 weeks old tomato plants although they found that the relative growth rate was not negatively affected.

## Concluding Remarks

Seed applied JA reduced feeding damage by western flower thrips but, among the eight evaluated tomato cultivars, silver damage symptoms were only reduced in JA-seed treated plants of the cultivar Carousel. The observed variability in responsiveness constitutes a major obstacle, because one of the foremost conditions for successful commercialization of elicitors is consistent efficacy. The variability among different cultivars clearly demonstrates the necessity to include multiple cultivars as a biological test system to establish consistent statements concerning compound efficacy. This is particularly important because, in scientific literature, Moneymaker along with tomato cultivars Aisla Craig, Micro-Tom and Castlemart are frequently used to represent tomato as a plant model system. Notably, we demonstrated that acid scarification significantly augmented responsiveness to seed applied JA in the non-responsive cultivar Moneymaker. This observation lends added support to the contention that permeability is an important factor for facilitation of JA absorption (i.e., bioavailability) and its’ exerted effect on defense responses. We conclude that future experiments focusing on seed structural properties, compound uptake as well as early transcriptional responses could provide explanations underlying the genotype dependent differences in seed permeability and, ultimately its importance in priming JA anti-thrips defenses. Nonetheless, the outcome of our work creates opportunities for tomato breeding programs to select for genetic traits that affect seed permeability as well as for private seed companies to pre-treat and manipulate nonresponsive cultivars.

## Data Availability Statement

The raw data supporting the conclusions of this article will be made available by the authors, without undue reservation.

## Author Contributions

SM, KL, and PK designed the experimental research. SM performed hormone seed and plant treatments and insect experiments. SM and IK performed (bio)-chemical analysis and interpretation. SM drafted the manuscript with critical review of all authors. All authors approved the final version of the manuscript.

## Conflict of Interest

The authors declare that the research was conducted in the absence of any commercial or financial relationships that could be construed as a potential conflict of interest.

## Footnotes

1 http://fiji.sc/Fiji

## Supplementary Material

Supplementary Figure 1 Effect of exogenous JA treatment on tomato resistance against WFT and JA-associated responses. **(A)** Silver damage symptoms in tomato plants (*n* = 12) following JA or mock treatment applied at the seed stage or as foliar spray 4 days prior to thrips infestation. Four weeks old tomato plants, variety Carousel, were subjected to a non-choice whole plant bioassay and infested with 20 adult thrips. Silver damage symptoms were visually scored after 7 days of infestation. **(B)** Polyphenol oxidase activity was measured in non-infested plants taking the third leaf from the bottom (*n* = 6–8). Data are presented as mean ± SEM. Different letters denote significant differences among groups as determined by GLM followed by Fisher’s LSD test (*P* < 0.05). The overall effects of application, treatment and their interaction are indicated in each graph.

Supplementary Figure 2 Effect of seed applied JA on dry mass of tomato plants. Dry mass was measured for 8 tomato cultivars; “Moneymaker,” “Carousel,” “72-323,” “Mahitos untreated,” “Mahitos Commercial,” “Brioso,” “Trovanzo,” and “Raymos” plants that were subjected to mock or jasmonic acid (3 mM) seed treatment. Data are means (+SEM) of 10 individual plants and different letters indicate significant differences among groups as determined by GLM followed by Fisher’s LSD test (*P* < 0.05). The overall effects of cultivar, treatment and their interaction are indicated in the graph.

Supplementary Figure 3 Effect of different pre-treatments on JA-induced defenses against WFT on silver damage and plant growth. Seeds were mechanically (razor blade), chemically (10% sulfuric acid) or commercially scarified prior to JA or mock seed treatment. Control seeds were non-scarified. **(A)** Total silver damage was evaluated in 4 weeks old tomato plants (cv. Virona) after 7 days of thrips infestation. **(B)** Dry mass was assessed as a measure of plant performance. Data are presented as mean ± SEM, *n* = 12. Different letters indicate significant differences among groups as determined by GLM followed by Fisher’s LSD test (*P* < 0.05). The overall effects of scarification method, treatment and their interaction are indicated in the graph.

Supplementary Figure 4 Effect of seed applied JA on tomato resistance against Wester Flower Thrips (*Frankliniella occidentalis*). Four weeks after seed treatment “Moneymaker” plants were subjected to a non-choice whole plant bioassay and infested with 20 adult thrips. Silver damage symptoms were visually scored after 7 days of infestation. Data are presented as mean ± SEM, *n* = 8. There was no statistically significant effect among treatments on silver damage symptoms [Welch’s *F*_(3, 15.19)_ = 0.36, *p* = 0.781].

Supplementary Table 1 Effect of jasmonic acid foliar application and seed soaking on plant growth parameters of tomato (cv. Virona). Data are expressed as mean ± SEM (*n* = 10).
