# Supplementary figures and images for: Cultivar Variation in Tomato Seed Coat Permeability Is an Important Determinant of Jasmonic Acid Elicited Defenses Against Western Flower Thrips
Source: Front Plant Sci. 2020 Nov 11;11:576505. doi: 10.3389/fpls.2020.576505 (PMC7686761; doi:10.3389/fpls.2020.576505)

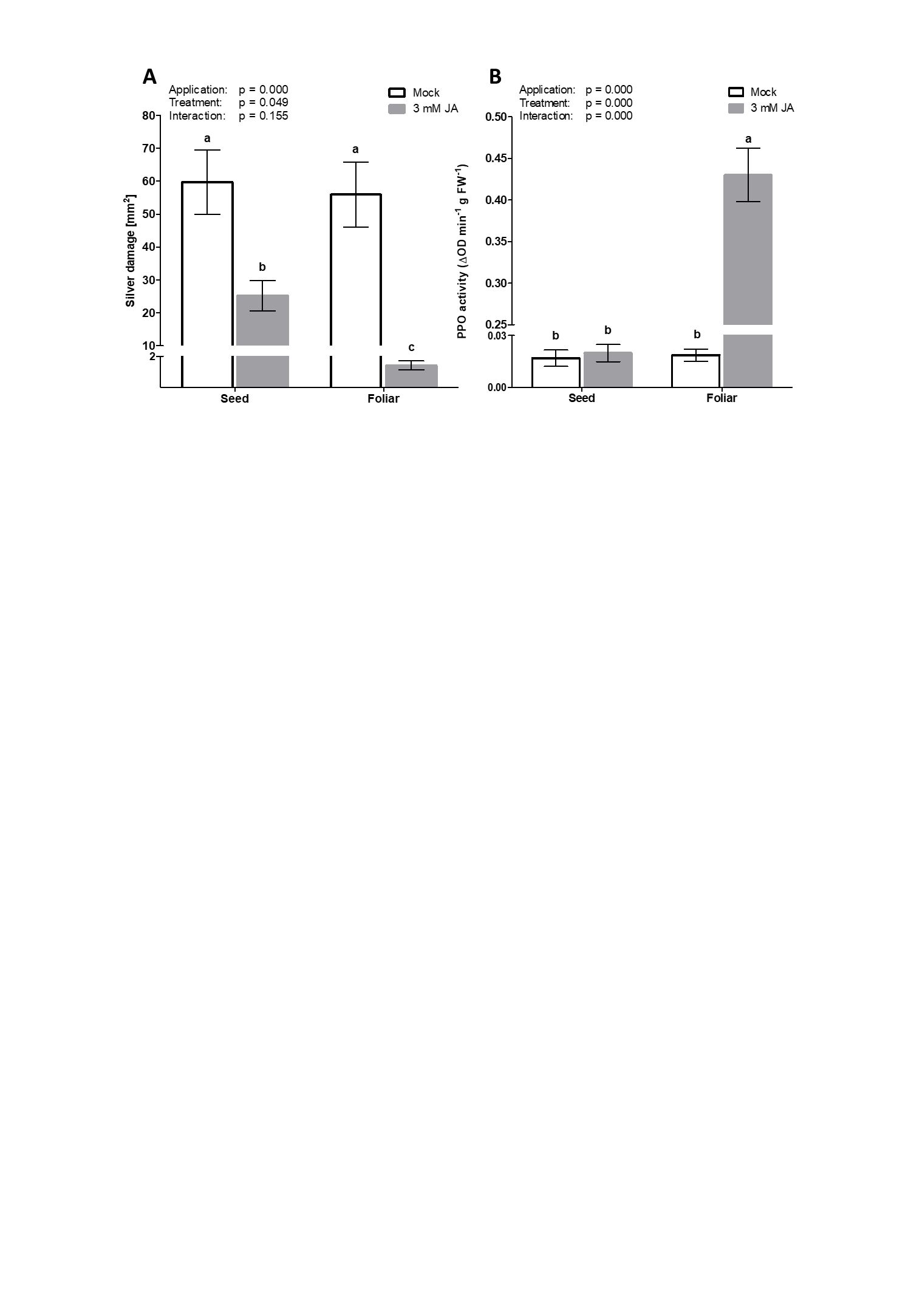

Supplement: Supplementary Figure 1 — Effect of exogenous JA treatment on tomato resistance against WFT and JA-associated responses. (A) Silver damage symptoms in tomato plants (n = 12) following JA or mock treatment applied at the seed stage or as foliar spray 4 days prior to thrips infestation. Four weeks old tomato plants, variety Carousel, were subjected to a non-choice whole plant bioassay and infested with 20 adult thrips. Silver damage symptoms were visually scored after 7 days of infestation. (B) Polyphenol oxidase activity was measured in non-infested plants taking the third leaf from the bottom (n = 6–8). Data are presented as mean ± SEM. Different letters denote significant differences among groups as determined by GLM followed by Fisher’s LSD test (P < 0.05). The overall effects of application, treatment and their interaction are indicated in each graph. [file Image_1.TIF]

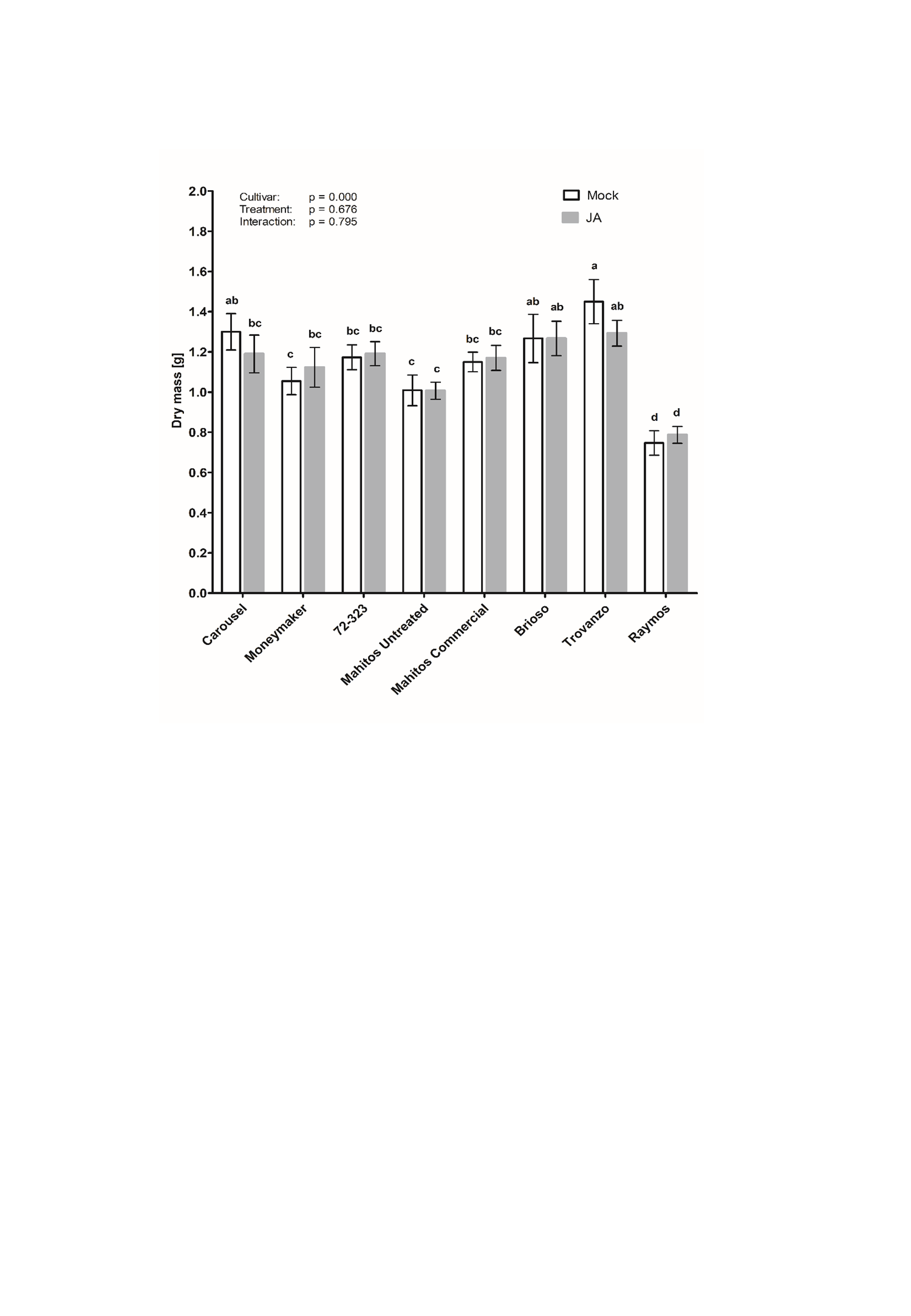

Supplement: Supplementary Figure 2 — Effect of seed applied JA on dry mass of tomato plants. Dry mass was measured for 8 tomato cultivars; “Moneymaker,” “Carousel,” “72-323,” “Mahitos untreated,” “Mahitos Commercial,” “Brioso,” “Trovanzo,” and “Raymos” plants that were subjected to mock or jasmonic acid (3 mM) seed treatment. Data are means (+SEM) of 10 individual plants and different letters indicate significant differences among groups as determined by GLM followed by Fisher’s LSD test (P < 0.05). The overall effects of cultivar, treatment and their interaction are indicated in the graph. [file Image_2.TIF]

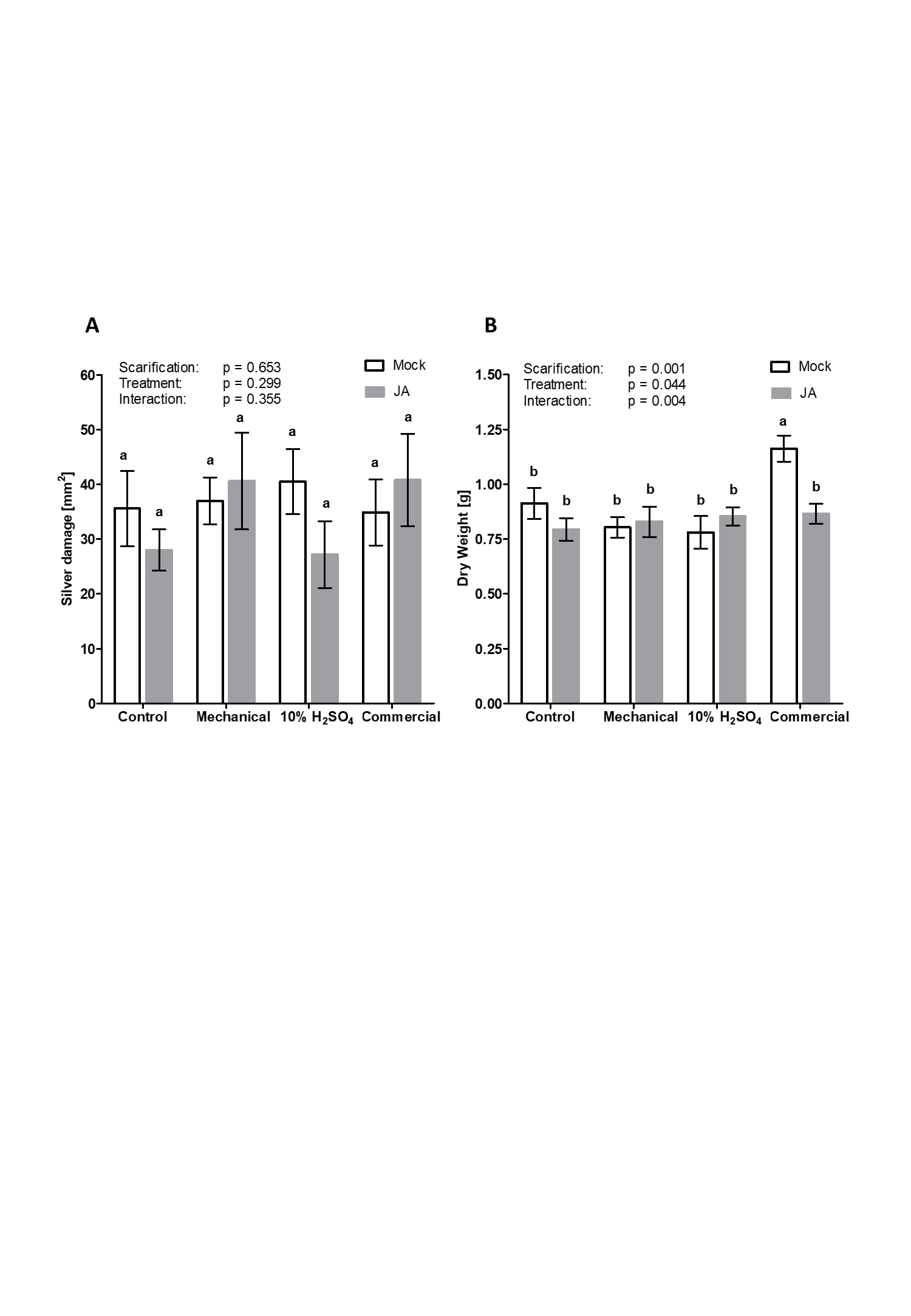

Supplement: Supplementary Figure 3 — Effect of different pre-treatments on JA-induced defenses against WFT on silver damage and plant growth. Seeds were mechanically (razor blade), chemically (10% sulfuric acid) or commercially scarified prior to JA or mock seed treatment. Control seeds were non-scarified. (A) Total silver damage was evaluated in 4 weeks old tomato plants (cv. Virona) after 7 days of thrips infestation. (B) Dry mass was assessed as a measure of plant performance. Data are presented as mean ± SEM, n = 12. Different letters indicate significant differences among groups as determined by GLM followed by Fisher’s LSD test (P < 0.05). The overall effects of scarification method, treatment and their interaction are indicated in the graph. [file Image_3.TIF]

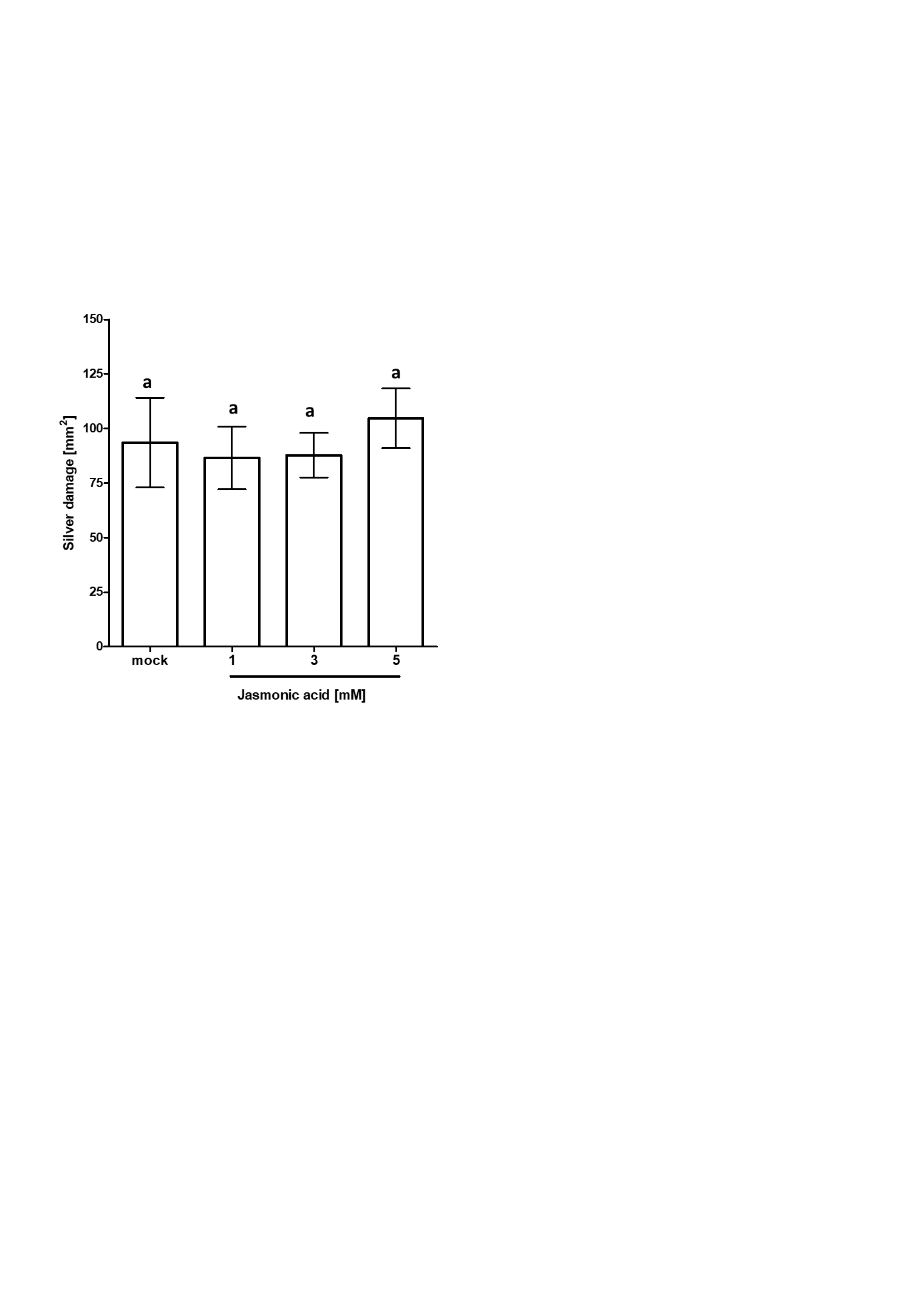

Supplement: Supplementary Figure 4 — Effect of seed applied JA on tomato resistance against Wester Flower Thrips (Frankliniella occidentalis). Four weeks after seed treatment “Moneymaker” plants were subjected to a non-choice whole plant bioassay and infested with 20 adult thrips. Silver damage symptoms were visually scored after 7 days of infestation. Data are presented as mean ± SEM, n = 8. There was no statistically significant effect among treatments on silver damage symptoms [Welch’s F(3, 15.19) = 0.36, p = 0.781]. [file Image_4.TIF]
