# Supplementary material for: Cultivar Variation in Tomato Seed Coat Permeability Is an Important Determinant of Jasmonic Acid Elicited Defenses Against Western Flower Thrips
Source: Front Plant Sci. 2020 Nov 11;11:576505. doi: 10.3389/fpls.2020.576505 (PMC7686761; doi:10.3389/fpls.2020.576505)
Supplement: Supplementary Table 1 — Effect of jasmonic acid foliar application and seed soaking on plant growth parameters of tomato (cv. Virona). Data are expressed as mean ± SEM (n = 10). [file Table_1.docx]

**Supplementary Table S1.** Effect of jasmonic acid foliar application and seed soaking on plant growth parameters of tomato (cv. Virona). Data are expressed as mean ± SEM (n=10).

|  | **Seed soaking** | | | **Foliar spray** | | |
| --- | --- | --- | --- | --- | --- | --- |
|  | No. Leaves^1^ | Plant Height^1^ | Dry mass^1^ | No. Leaves^2^ | Plant Height^1^ | Dry mass^1^ |
| Mock | 6,3±0,15^a^ | 11,96±0,64^abc^ | 0,37±0,03^cd^ | 6,0±0,15^a^ | 13,61±0,41^a^ | 0,63±0,05^a^ |
| 0,1 mM JA | 6,3±0,21^a^ | 11,37±0,42^bcd^ | 0,39±0,03^bc^ | 5,9±0,18^a^ | 14,96±0,69^a^ | 0,62±0,04^a^ |
| 0,5 mM JA | 6,3±0,11 ^a^ | 11,10±0,35^cd^ | 0,33±0,01^cd^ | 5,8±0,13^a^ | 14,44±0,77^ab^ | 0,59±0,04^ab^ |
| 1 mM JA | 6,4±0,16 ^a^ | 13,29±0,55^a^ | 0,50±0,04^a^ | 5,5±0,22^ab^ | 12,53±0,41^b^ | 0,50±0,03^b^ |
| 3 mM JA | 6,2±0,20 ^a^ | 12,77±0,53^ab^ | 0,47±0,04^ab^ | 4,8±0,20^b^ | 9,36±0,48^c^ | 0,34±0,04^c^ |
| 5 mM JA | 6,2±0,13 ^a^ | 10,39±0,65^d^ | 0,28±0,03^c^ | 4,5±0,17^b^ | 7,93±0,26^c^ | 0,33±0,04^c^ |

Data followed by different letters indicate significant differences among treatment means in a column tested by Fisher’s LSD^1^ or Dunn’s test^2^ at *P* <0.05.
